# Lymph Node-on-Chip Technology: Cutting-Edge Advances in Immune Microenvironment Simulation

**DOI:** 10.3390/pharmaceutics16050666

**Published:** 2024-05-16

**Authors:** Qi Wang, Yuanzhan Yang, Zixuan Chen, Bo Li, Yumeng Niu, Xiaoqiong Li

**Affiliations:** Beijing Key Laboratory for Separation and Analysis in Biomedicine and Pharmaceuticals, School of Medical Technology, Beijing Institute of Technology, Beijing 100081, China; 3120211989@bit.edu.cn (Q.W.); 3120215950@bit.edu.cn (Y.Y.); zx-chen@bit.edu.cn (Z.C.); boli@bit.edu.cn (B.L.); yumengniu928@gmail.com (Y.N.)

**Keywords:** lymph node, lymph node-on-chip, lymph nodes microenvironment, in vitro models

## Abstract

Organ-on-a-chip technology is attracting growing interest across various domains as a crucial platform for drug screening and testing and is set to play a significant role in precision medicine research. Lymph nodes, being intricately structured organs essential for the body’s adaptive immune responses to antigens and foreign particles, are pivotal in assessing the immunotoxicity of novel pharmaceuticals. Significant progress has been made in research on the structure and function of the lymphatic system. However, there is still an urgent need to develop prospective tools and techniques to delve deeper into its role in various diseases’ pathological and physiological processes and to develop corresponding immunotherapeutic therapies. Organ chips can accurately reproduce the specific functional areas in lymph nodes to better simulate the complex microstructure of lymph nodes and the interactions between different immune cells, which is convenient for studying specific biological processes. This paper reviews existing lymph node chips and their design approaches. It discusses the applications of the above systems in modeling immune cell motility, cell–cell interactions, vaccine responses, drug testing, and cancer research. Finally, we summarize the challenges that current research faces in terms of structure, cell source, and extracellular matrix simulation of lymph nodes, and we provide an outlook on the future direction of integrated immune system chips.

## 1. Introduction

Lymph nodes (LNs) are integral components of the immune system, functioning as critical sites for detecting foreign antigens within the lymphatic fluid and facilitating the orchestration of intricate immune cell interactions [1,2]. The extracellular matrix (ECM) within LNs provides structural support for resident immune cells. It plays a regulatory role in their migratory patterns and functional capacities, maintaining homeostatic fluid balance and efficient cellular trafficking throughout the organism [3,4,5,6]. Anatomically, LNs are delineated into several distinct regions: an encapsulating outer layer, a cortex predominantly populated by B lymphocytes, and a medullary region where T lymphocytes and plasma cells reside [1,7,8,9]. These specialized cellular contingents engage in a synergistic interplay, crucial for recognizing and neutralizing pathogens through filtration of lymphatic fluid, which is pivotal in eliciting robust immune responses [10,11,12]. Dysfunctions in lymphocyte activities, such as atypical T lymphocyte activation, uncontrollable B lymphocyte proliferation, and impaired natural killer (NK) cell functionality, may precipitate the onset of a spectrum of disorders encompassing autoimmune diseases [13] and an increased vulnerability to viral infections [14]. Consequently, an exhaustive understanding of the lymphatic system’s complex functions and specific immunological outcomes is essential for meticulously evaluating immune cell responses to therapeutic interventions, the precision of disease diagnostics, and the development of targeted treatment modalities [15,16].

**Figure 1:**
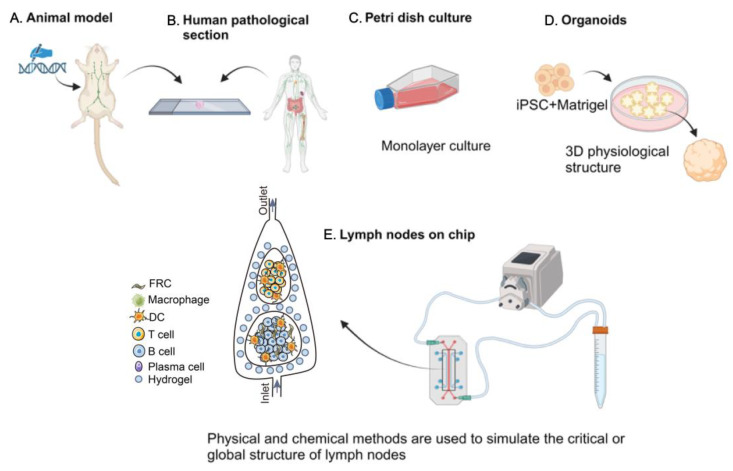
Advancements in lymph node research models. (**A**) Animal model construction and characterization. (**B**) Human tissue and organ preparation for pathological section observation. (**C**) Extracted mouse lymph node cells in suspension culture. (**D**) Organoid model. Induced pluripotent stem cells extracted from the human body are mixed with matrix gel to prepare a three-dimensional spherical structure organoid. (**E**) Organ-on-a-chip model. PDMS, hydrogel, and other raw materials are used to construct and simulate the structure of LNs, into which the corresponding cells of lymph node tissues are added. Peristaltic pumps, tubes, and other materials are integrated to create a closed-loop system that accurately replicates the dynamic flow conditions within the human body. (created by Biorender).

Traditional research models include suspension cell cultures, animal models, human pathological tissue and organoid studies. The utilization of rodent animal models, such as mice and rats, provides a valuable platform for studying the complex environment within the human body, particularly in understanding the structure, function, and immune response mechanisms of LNs [17,18,19]. These models offer a robust physiological framework for researchers to investigate various aspects of LNs biology in vivo [20]. Using specific antigen stimulation or gene editing techniques, researchers can effectively simulate pathological states such as infection and inflammation in these animal models, after which mouse LNs are extracted and pathological sections are made to observe the corresponding pathological changes, as shown in Figure 1A. In addition, animal models can also be evaluated using cell flow cytometry and microscopy to assess the effects of drugs or vaccines on lymph node function [18]. For instance, a recent study has supported the notion that a slow-release immune strategy can enhance germinal center responses and neutralize antibody (Ab) generation, providing a promising avenue for vaccine design [21]. However, it is essential to recognize the inherent interspecies differences between mice and humans that may impede the direct translation of research findings from animal models to human biology. Discrepancies in the immune system, anatomical structure, cellular composition, and immune response characteristics of LNs between mice and humans may limit the generalizability of results [22,23]. To overcome this limitation, researchers have introduced humanized mouse models, where human hematopoietic stem cells are engrafted into immunodeficient mice, creating animal models with human immune system characteristics [24,25]. This innovative approach presents a promising platform for investigating human-specific LNs immune responses; however, it still falls short of fully capturing the intricate milieu within the human body [26]. However, the main drawbacks of current humanized mice are either the presence of a type of transplant rejection [27], the lack of thymus leading to a lower level of human T cell development, or the restricted source of donors and the complexity of model construction [28]. The sample size of tissues and organs directly derived from the clinical human body is very scarce, and the data source is also histopathological section observation and analysis, as shown in Figure 1B.

Ex vivo culture methodologies, including LNs fragment cultivation and cell suspension culture modalities, have been widely employed to recapitulate the cellular composition and immunological microenvironment of lymphoid tissues [20,29]. These advanced techniques allow for the dissociation and subsequent isolation of immune effector cells from the lymphoid organs of humans or model organisms, enabling the investigation of the diverse functional properties and differentiation pathways of various leukocyte subsets, such as T lymphocytes, B lymphocytes, and dendritic cells (DCs), under controlled laboratory conditions [30]. Notably, intricate co-culture systems juxtaposing T lymphocytes with antigen-presenting DC have provided profound insights into the complex molecular cascades governing T cell activation and lineage commitment within the LNs architecture, Figure 1C. Traditional two-dimensional cell culture techniques often face inherent limitations, especially the inability to replicate complex three-dimensional structures and microenvironmental features of lymphoid tissues, which may lead to experimental results that deviate from the physiological in vivo state [31,32]. Concurrent with the advancement of three-dimensional culture technologies, researchers have increasingly employed biocompatible matrices or cellular aggregates, known as spheroids, to create experimental constructs that more accurately mimic the structural and functional intricacy of human lymphoid organs, as in Figure 1D [33]. Groundbreaking progress has highlighted the potential of combining biodegradable scaffolding materials with resident LNs stromal cell populations in co-culture to reconstitute three-dimensional configurations that closely resemble native lymphoid organoids. This technological renaissance has enhanced our understanding of the migratory patterns and interaction strategies within lymphoid tissues and established a more relevant platform for investigative pursuits [34,35]. The inherent advantage of in vivo culture systems lies in the ease of experimental manipulation and the precision with which conditions can be modulated, facilitating the detailed examination of specific cellular entities or molecular pathways [36].

Organ-on-a-chip technology, leveraging the remarkable progress in microfluidics, biomaterials, and tissue engineering, has emerged as a groundbreaking platform for simulating human tissues and organs in vitro, offering unprecedented opportunities for drug screening, disease modeling, and particular medical exploration [37,38,39]. The primary functions of human organs are simulated by assembling cultured cells into three-dimensional (3D) tissue structures. The core objective is to create a controllable minimal functional unit that mimics the physiological state of the human body [40]. In contrast to traditional animal models, lymph node chips can overcome animal species differences by using human-derived cells to construct physiological unit structures to perform specific functions [41]. Cells cultured in vitro in petri dishes do not have 3D tissue or organ structures and cannot mimic in vivo physiological structures [42]. Compared to organoids, chips can be derived from a wider variety of cell sources, either from cell lines or from human primary cells, and utilize advanced technologies to reproduce specific functions within the lymph node. In addition, a microfluidic system can better simulate the flow state of body fluids, which is closer to the in vivo physiological environment [43]. This innovative approach enables more precise observation and investigation of biological processes and a more accurate assessment of drug safety and efficacy than conventional tissue culture methods and animal models [44]. Compared to static model-based approaches (e.g., organoids based on hydrogel scaffold), organs-on-a-chip can more closely mimic the natural physiological microenvironment and associated immune functions under the dynamics of lymphatic flow, thus more accurately replicating physiological dynamic conditions in vivo [9], as shown in Figure 1E. In addition, lymph node-on-chip allows for fine control and study of critical parameters of the lymph node system, such as shear stress (i.e., dynamic mechanical stress), continuous fluid flow, distribution of drugs/molecules, concentration gradients, and high cell densities, and have become powerful tools in the fields of pharmacology and toxicology in recent years [20,45]. The development of lymph node chips has promoted research on the interaction mechanism between immune cells and drug candidates, which is expected to reduce the high cost and high failure rate of drug development [46,47,48]. This innovative approach allows for more precise observation and study of biological processes, as well as more accurate assessment of drug safety and efficacy, compared to traditional tissue culture methods and animal models. [39].

This review meticulously examines the evolution and recent innovations in material science, compositional strategies, and structural design of chip-based systems that emulate the lymph node milieu in vitro. It discusses vital research directions in utilizing these chip models and their implications in pathology and pharmacology. Furthermore, the paper explores the potential developments of lymph node chip technology. The review provides a comprehensive understanding of how this technology has transformed our comprehension of immune system functions and associated diseases. It analyses the progression and key breakthroughs in lymph node chip technology, especially its ability to mimic the immune microenvironment. Thus, this critical evaluation underscores the importance of lymph node chip technology as a novel approach in immunological investigations, offering valuable insights and methodologies for future research in immunology and the treatment of immune-related disorders.

## 2. Integrating Lymph Node Models with Organ-on-a-Chip

The development of lymph node chips entails a complex design process that includes creating a biomimetic scaffold suitable for seeding and supporting immune cells, replicating the natural ECM microenvironment, simulating the dynamic changes in lymphatic flow seen in living systems, and faithfully reconstructing the unique cellular organization and spatial layout typical of LNs [49,50,51]. Researchers have used various methods to construct these advanced in vitro models of lymph node structures (Table 1).

### 2.1. Design and Fabrication of Lymph Node-on-Chip Technology

#### 2.1.1. Lymph Node-on-Chip Scaffold Material

**Figure 2:**
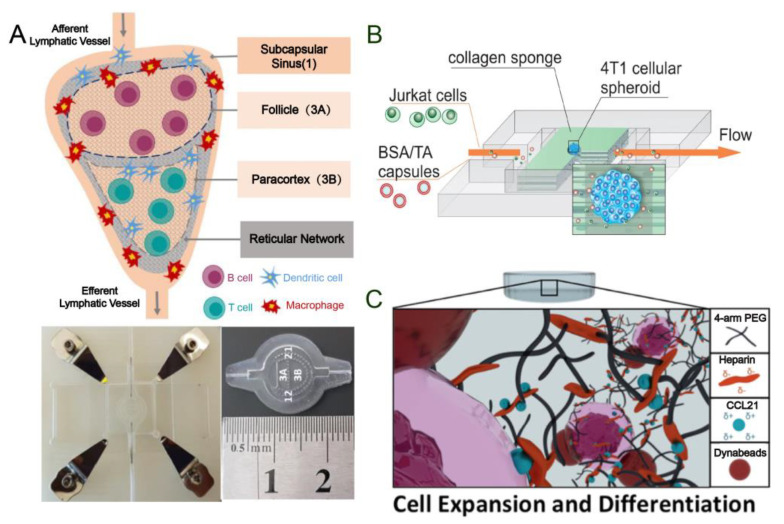
(**A**) Pattern diagrams and micrographs of microchips of simulated human LNs made from PDMS [59]. Reproduced with permission from Aya Shanti, Pharmaceutics; published by MDPI 2020. (**B**) A chip model using collagen sponges to mimic the cytoplasmic matrix of LNs and to study secondary tumors in lymphoid tissue [33]. Reproduced with permission from Sergei V. German, International Journal of Molecular Sciences; published by MDPI 2023. (**C**) Schematic diagram of a lymph node chip using a hydrogel formed by combining low molecular heparin with polyethylene glycol to mimic a cytoplasmic matrix and study T cell proliferation [78]. Reproduced with permission from Eduardo Pérez del Río, Biomaterials; published by ELSEVIER 2020.

The agarose gel wall offers an adequate physical barrier for convective fluid flow and it separates the flow control unit from the matrix-containing cells. Additionally, agarose can provide a stable scaffold for common ECM components, such as fibrin and collagen [63,64]. Agarose gel can also uncouple the inherent coupling between fluid flow and chemical concentration gradients. Currently, there are 3D microfluidic devices that use agar to establish chemokine gradients. These devices are used to study the response of DC to chemokine gradients and how CCL19 and CCL21 produce different signals to regulate the homing and localization of DC in the lymphatic system [52,53]. Since agarose gel has a network structure, small molecules experience less resistance when migrating, making it easier to study the migration tendency. However, large molecules encounter more resistance during their motion. Agarose has poor mechanical strength and breaks easily [65], as a common carbon source for bacteria is prone to bacterial contamination [66]. Polydimethylsiloxane (PDMS) is a popular material for organ-on-chip fabrication, with microfluidic channels often easily formed through soft lithography [67]. It is known for its biocompatible, hydrophobic, and stable chemical properties in perfusion systems. In combination with hydrogel materials, it is possible to set specific concentrations to mimic the concentration of chemokines in the LNs to explore the migration of the relevant immune cells. It also facilitates the construction of regionalized lymph node structures (Figure 2A) [59]. However, there are still some shortcomings when using PDMS materials to fabricate microfluidic chips, such as a higher number of required steps, low throughput, and the absorption of small molecules. Follow-up studies are needed to refine the automation of microchip fabrication to compensate for the shortcomings. Polymethylmethacrylate (PMMA) belongs to rigid organic polymer materials, also known as acrylic or plexiglass, which has the advantages of high transmittance, biocompatibility, and low cost, and PMMA is stronger than glass [68]. Despite these advantages, PMMA has limitations in processing accuracy, making PDMS the optimal choice for specific finishing requirements.

#### 2.1.2. Simulation of ECM

The most commonly used chip culture hydrogel is made of collagen, fibrinogen, matrix glue, gelatin, polyacrylamide (PA), polyethylene glycol (PEG), or hyaluronic acid (HA) [69]. Type I collagen is the primary component of the ECM in LNs [70]. Most lymph node chips employ type I collagen-based hydrogels to mimic the lymph node ECM microenvironment. Furthermore, fibronectin can bind to various proteins such as proteoglycans, growth factors, integrins, and chemokines. Therefore, it is frequently used to reconstruct the ECM [55,58,71]. The chemical properties, composition, porosity, stiffness, cross-linking, and topological structure of hydrogels are crucial factors that influence immune cell migration and can impact the in vitro modeling of immune cell migration [72,73]. For example, Sergei et al. developed a microfluidic lymph node-on-chip (LNOC) to mimic the morphology and porosity of human lymph node ECM through a collagen sponge. The parameters of the LNs model can be more accurately adjusted by altering the shape and pore size of the collagen sponge [33]. (Figure 2B) The pores or openings in the ECM, also known as interstitial spaces, can vary in size and shape depending on the location and the state of the lymph node. In this research, they used scanning electron microscopy (SEM) to analyze collagen and pore size and distribution. Generally speaking, the pore diameter in normal LNs ranges from 20 to 50 μm [34]. The obtained results are within the above range. To simulate a more realistic ECM of LNs in vivo, recent studies have combined stromal cells with hydrogels [74,75]. Research has demonstrated that enclosing stromal cells in fibrin, collagen, or fibrin–collagen hydrogel can enhance the creation of a matrix network and facilitate T cell infiltration [76]. Primary fibroblastic reticular cells (FRC) were introduced in vitro in the collagen–fibrin hydrogel model of cultured dendritic cell lines. FRC has been shown to enhance DC function in a more realistic human lymphatic microenvironment [77]. Perez et al. have designed a 3D PEG hydrogel that is covalently bound to low molecular weight heparin to mimic a lymph node for the proliferation of T cells (Figure 2C). PEG provides the necessary structural and mechanical properties, while heparin anchors CCL21. The 3D structure and load capacity of hydrogels can enhance primary human CD4^+^ T cell proliferation [78]. The ideal biomaterial should possess comparable stiffness, degradability, and capacity to support soluble factor binding, cell adhesion, communication, and movement as the target tissue [79,80]. We can then construct organ chips that more closely resemble LNs in terms of their ECM environment in vivo. These models can be used to study the complex interactions between stromal cells and other immune cells, including immune cell chemotaxis and migration, as well as the immune response of stromal cells in vitro.

Researchers will further characterize lymph node models to reflect a replication of the in vivo lymph node microenvironment. For example, the Cesare team characterized the collagen matrix of the multicompartmental lymph node chips they developed. Specifically, reconstruction of the topological parameters of the substrate using a customized image processing toolbox revealed that the collagen network had a porosity of 74.35 ± 2.19%, a pore size of 3.973 ± 0.460 μm, and that the structure of the collagen substrate displayed a protofibrillar morphology. Using the developed experimental setup, collagen permeability was measured to be 3.84 × 10^−15^ ± 2.41 × 10^−16^ m^2^ by Darcy’s law; matrix elasticity was quantified using the ElastoSense Bio2, with a stiffness of 138.62 ± 1.54 Pa, which is within the range of human LNs stiffnesses [81].

Several studies have shown that fluid flow generated by mechanical forces is similar to fluid flow in the body and can affect cells’ shape, function, interaction, and differentiation process [82,83,84]. The flow dynamics within the LNs and how its internal structure affects lymphatic fluid flow are critical to the immune response [9]. The lymph node chip achieves reasonable flow rate control through an active pumping system to simulate in vivo fluid dynamics. Moura et al. used an injection pump to continuously perfuse and control the flow rate to detect the dynamic interaction between flowing lymphocytes and adherent dendritic cells under five orders of magnitude of shear stress (from 0.01 to 100 Dyn/cm^2^) in real time. This provides the basis for studying the dynamics of cell–cell interactions in different biological environments related to the adaptive immune system, including variable speed, shear stress, deformation rate and migration processes [55]. In another study, the COMSOL model was first used to predict the wall shear stress (WSS) curves of LNs under static and inflammatory conditions. In a microfluidic device simulating the subcapsular sinus microenvironment, the channel width of the divergent part of the functionalized adhesive molecules increases linearly. Two types of WSS curves were reproduced by designing divergent shapes and selecting perfusion parameters used to investigate the effect of lymph node remodeling on cell adhesion in fluid flow associated with lymph node metastasis [62].

#### 2.1.3. Unique Cell Source of Lymph Node-on-Chip

Limited antigen specificity may limit their ability to recognize more than one antigen in immune chip models [85]. In most studies, only one or two types of immune cells have been used in their lymph node devices. The sources of cells are mainly divided into two categories. The first category is the immortalized human cell line. Common DCs are derived from the polarization of the mutz3-1 cell line, and the common T and B cell lines are Jurkat T cells and Raji B cells [86,87]. The second type consists of multicellular suspensions extracted from peripheral blood and single-cell suspensions after cell sorting. Some researchers also directly selected lymph node slices and added them to the chip to explore the function of LNs [88,89]. Cell lines are mature models that can be easily modified to target and study molecules of interest. However, immortalized cells cannot fully reproduce primary cells’ morphological and functional characteristics. The use of primary immune cells, which are typically purified from human blood or lymph node tissue, can overcome this limitation. While tissue slices can preserve the original spatial tissue and matrix network of organs, they are not often obtained from patients. Blood-derived lymphocytes are readily available and provide a rich source of cells, including T cells, B cells, granulocytes and monocytes in different activated states (such as naive, effector and memory T cells) [90], but require purification before use in the model system. Additionally, immune cells derived from the patient’s blood are specific and have the potential for personalized medical treatment.

#### 2.1.4. Replication of the Spatial Configuration of the Lymph Node

**Figure 3:**
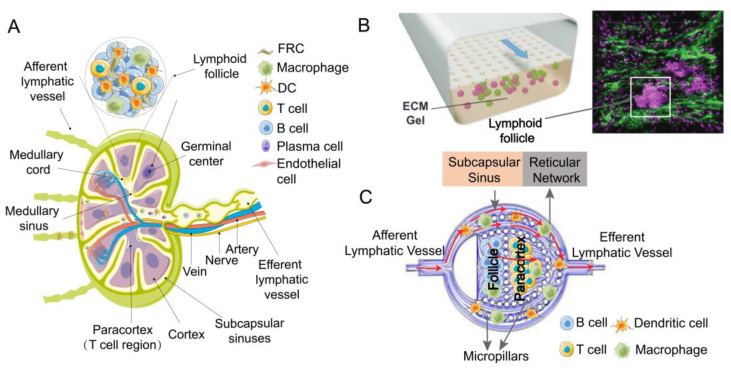
(**A**) Physiological structure of LNs. (**B**) Two-channel lymph node organ-on-a-chip model [61]. Reproduced with permission from Girija Goyal, Advanced Science; published by Wiley-VCH GmbH 2022. (**C**) Biomimetic localized lymph node chip [59]. Reproduced with permission from Aya Shanti, Pharmaceutics; published by MDPI 2020.

The lymph node constitutes a sophisticated framework wherein diverse cell populations are strategically distributed. Authentic LNs delineate a B cell zone, delineated by inwardly protruding trabeculae and lymphoid follicles (LFs), comprising a network orchestrated by follicular dendritic cells (FDCs) [91]. The paracortical zone, also integral, harbors a concentration of T cells and DCs, constituting the T cell zone, crucial for T cell priming through the capture of antigens by DCs in the periphery, followed by migration into LNs [92] (Figure 3A). Notably, replicating the spatial intricacies of LNs poses a formidable challenge due to the intricate cellular distributions. Presently, membrane filtration stands as the primary method for bifurcating two channels: one establishes a chemokine concentration gradient to foster DC expansion or induce the formation of T and B cell follicular centers, while the other channel facilitates immune cell chemotaxis through continuous perfusion of culture medium laden with immune cells or antigens (Figure 3B). This encompasses bioassay and imaging to scrutinize migration, adhesion, and interactions among immune cells and antigen-presenting cells (APC) [55,57,58,61]. Precisely emulating the co-culture and spatial arrangements of diverse cell types, akin to their in vivo counterparts, remains a pivotal challenge in vitro lymph node modeling. Presently, most in vitro lymph node chips falter in replicating the compartmentalized structure observed in live LNs. Cesare’s team has pioneered the development of a multi-chambered 3D lymph node chip that encapsulates the essential attributes of natural human LNs [59]. The chip has a compartmentalized structure similar to LNs in the body. The upper region corresponds to the follicles housing B cells, while the lower region aligns with the paracortical area where T cells reside. This model impeccably mirrors the physiological compartmentalization of LNs, achieved by strategically using hydrogel-based microcolumns to segregate crucial locations and facilitate cell inoculation in distinct areas (Figure 3C). Such meticulous compartmentalization supports prolonged cell culture and replication of lymphatic flow patterns. Consequently, it has been instrumental in evaluating the impact of drugs on T and B cells [60]. Nonetheless, there are still limitations to this model. Notably absent are stromal cells and additional types of lymph node immune cells. Furthermore, the reliance on cancer-derived immune cell lines underscores the need for incorporating human-derived cells in future iterations of lymph node organ chips.

### 2.2. Research Insights Derived from Lymph Node-on-Chip Technology

#### 2.2.1. Chemokines Facilitate the Migration and Homing of Immune Cells

Preliminary studies using lymph node microfluidic devices have focused on characterizing cell migration. Chemokines secreted by stromal cells mediate the localization of immune cells from the external region of the antigen to the correct compartment of immune activation in LNs and provide a scaffold for cell migration [93]. DCs present pathogens or tumor-exposing antigens that stimulate T and B cells in lymphoid tissues, leading to an immune response at the site of the lesion. Chemokines, such as CCL19, CCL21, and CXCL12, play a vital role in the immune response triggered by DCs. Haessler et al. used an agarose-based 3D microfluidic chemotaxis device to quantify the chemotaxis of mouse DC to a CCL19 gradient. This device is suitable for studying rapidly migrating cells, which is conducive to studying 3D chemical invasion of cancer cells or immune cells [53]. Ricart et al. reproduced the chemotaxis of single and competitive chemokine gradients in mouse bone marrow-derived DCs using another microfluidic device. They found that the CCL19 signal was stronger than that of CCL21 or CXCL12 [54].

Lin et al. used a fibronectin-coated Y-type microfluidic device to study the chemotaxis of blood-derived T cells to single and competitive gradients [56]. CCL21 and CCL19, which are CCR7 ligands, work together to transport and localize T cells in lymphoid tissues, which is crucial for immune surveillance and response. To investigate their interaction, the Lin team used a Y-type fluid device to study the effect of coexisting concentrations of CCL19 and CCL21 on guiding T cell migration in vitro. The results were explained through a mathematical model and computer simulation. Human peripheral blood T cells were found to migrate toward CCL21 rather than CCL19 under physiological conditions. Sonmez et al. developed a flow-free chemokine gradient chip system and used a stable gradient to control Jurkat cell chemotaxis and chemical kinetics. The results showed that the intensity of Jurkat cell chemotaxis in response to the CXCL12 gradient decreased with increasing surface fibronectin and that the concentration and gradient of the CXCL12 chemokine affected Jurkat cell chemotaxis. It is also possible to study the cross-regulation effects of multiple factors on cell movement in different biological processes [58].

Migration of B cells within LNs is essential for an adaptive immune response. Chemotaxis gradients are believed to direct B cells to move into the follicle, then move to specific areas of the follicle during activation, and ultimately exit [94]. Chemokine CXCL13 is critical in directing B cells to secondary lymphoid organs and germinal centers (GCs) [95]. Another chemokine, CXCL12, initially attracts immature B cells to the dark area of the germinal center, where they proliferate and interact with FDCs, after which B cells lose expression of the CXCL12 receptor CXCR4 and follow the CXCL13 gradient to the light area of the germinal center, where somatic hypermutation occurs [96]. However, there is no in vitro lymph node-on-chip to simulate the migration and transformation of B cells.

#### 2.2.2. Interaction between T Cells and APCs in the Paracortical Region

**Figure 4:**
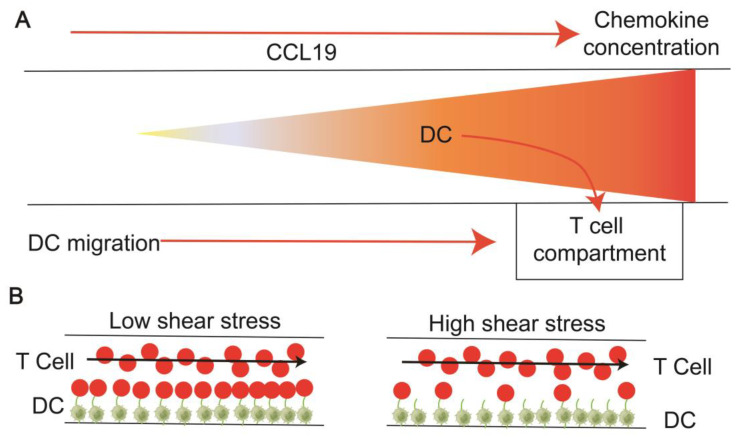
Diagram modeling the interaction between T cells and APCs. (**A**) DCs converge to T cell compartments in the presence of chemokines [57]. (**B**) T cells bind to DCs under the influence of different shear stress [55].

In response to inflammatory signals, APCs acquire local antigens and migrate to LNs, where they bind to T cells for adequate antigen recognition and trigger an adaptive immune response. Understanding how T cells migrate and communicate in the LNs is vital to facilitate immunotherapy or vaccine design [97,98]. Researchers have simulated different in vitro models of LNs to study the interaction between T cells and DCs. One researcher built a two-layer PDMS microfluidic device in vitro. The top layer is a chemotaxis chamber where DCs are subjected to a chemokine gradient for migration. DCs undergoing chemotaxis settle into the T cell compartment below the chemotaxis chamber due to gravity, and the rapid DCs-induced activation of T cells is assessed by measuring calcium levels in the T cells (Figure 4A). The results show that DCs respond to chemokine concentrations and cross chemotactic channels without suffering damage, retaining the ability to activate T cells. This device can comprehensively assess DC chemotaxis and T cell activation induced by migrating DCs [57]. In vivo, T cell–APCs interactions occur at different interstitial flow rates. To further understand the intercellular dynamics and physiological conditions in the LNs, the researchers constructed a microfluidic device to observe the effects of flow shear on intercellular interactions. The platform is a PDMS-based biochip placed on a glass subplate and consists of a main flow channel with two inlets and two outlets. Two channels flowed continuously to introduce antigen-specific T cells, which migrated to the antigen-presenting DCs monolayer of the main channel, respectively, and the antigen-specific and non-specific attachment and detachment of CD8^+^ T and CD4^+^ T cells to DCs under different shear stresses were observed (Figure 4B). At a physical shear stress of 0.01 dyn/cm^2^, the average duration of T cell–APCs interaction was 12.8 min. This duration and the intermittent migration of neighboring DCs are consistent with those observed in vivo, demonstrating the effect of shear stress on T cell induction and activation. This model opens new possibilities for reconstructing the threshold of controlled mechanical force for immune cell receptor binding [55].

#### 2.2.3. B Cell Follicular and Germinal Center Model

Currently, the experimental model of human humoral response in the lymph node microenvironment in vivo and in vitro has not fully replicated the complexity of the process of B cell differentiation and affinity maturation in the germinal center reaction. Upon antigen presentation by APCs or direct antigen contact, naive B cells differentiate into Ab-secreting plasma cells, memory B cells, and long-acting plasma cells through the germinal center reaction. This process is divided into cloning and amplification, somatic hypermutation, and switch-like recombination [99]. Reproducing B cell interactions help to probe how LNs mediate adaptive immune responses to infections and vaccines [100].

Based on three-dimensional biomaterials, the B cell follicular organoid system is primarily used to investigate how the lymphatic microenvironment influences the germinal center reaction and subsequent Ab production. For instance, applying maleimide (mal)-based functionalized PEG in designing immune tissues can regulate B cell differentiation and enhance the population of antigen-specific germinal center B cells when similar T cell signals are present [101]. Braham et al. have developed a synthetic polymer-based 3D lymphoid model incorporating human B cells in a PEG-4MAL hydrogel, supported by CD40L cells, human tonsil-derived lymphoid stromal cells and cytokines, and optimized for human B cell culture. This model better encapsulates the tissue complexity of the germinal center and enhances in vitro B cell survival, proliferation, differentiation, and Ab production [36]. In their recent study, Goyal et al. created a microfluidic organ chip replicating human LFs in vitro. The platform consisted of two channels separated by a porous membrane. Primary human B and T cells isolated noninvasively from peripheral blood were cultured in an ECM gel consisting of matrix gel and type I collagen in the lower channel. In contrast, the upper channel provided oxygen and nutrients to the cells through a continuous perfusion channel. Under perfusion conditions, T and B cells spontaneously self-assemble into 3D LFs and undergo Ab class switching to form clusters of plasma cells, which secrete antigen-specific IgG upon antigen stimulation after adding 2% DCs [57].

**Figure 5:**
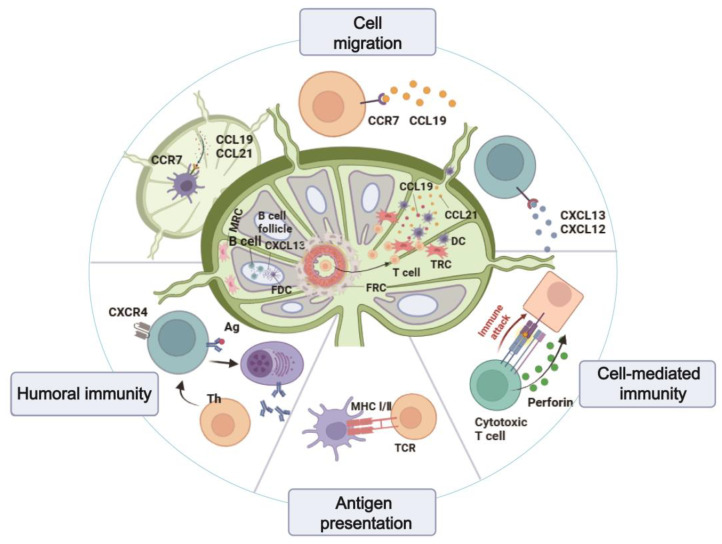
Lymph node chips’ main research directions. Includes chemokine-mediated migration and homing of immune cells, antigen presentation, cellular immunity, and humoral immune activation of GCs. (Created by Biorender).

Chemokines regulate the migration and localization of immune cells, and their homeostasis is crucial for maintaining lymph node structure. In the LNs and spleen, chemokines regulate the adaptive response by facilitating the initial activation of lymphocytes and guiding their differentiation and phenotype [102]. Pathological induction or inappropriate gradient of soluble signaling molecules can lead to the development of immune deficiency diseases, autoimmune diseases, chronic inflammation, and cancer metastasis [58,103]. Understanding gradient sensing can be used to develop new treatments for diseases, for example, localized drug delivery for the immunomodulation of inflammatory diseases [104] and T cell engineering for cancer immunotherapy [105]. The emergence of the chip model of T cell–APCs interaction and dynamic microenvironment changes in the LNs paracortical region shows the potential mechanism of T cell initiation, activation and proliferation in the immune response process in vitro. This study lays a foundation for follow-up studies of the temporal and spatial changes in LNs in response to inflammation and disease, antigen binding, vaccination, and tumor drainage [106]. In LNs, antigen-specific B cells in LFs trigger humoral immunity when they acquire antigens from the lymph fluid diffused from the subcapsular sinus. After APCs present or encounter antigens directly, naïve B cells differentiate into Ab-secreting plasma cells, memory B cells, and long-lived plasma cells through germinal center reactions. These cell populations initiate a systemic Ab-driven infection response by reducing pathogen transmission and providing lasting protection after vaccination [107]. Research and development of lymph follicular and germinal center on-chip will further explore the adaptive immune response to infection and vaccine in LNs, advancing vaccine development and preclinical detection (Figure 5).

## 3. Applications of Lymph Node on-a-Chip

### 3.1. Immune Response of Lymph Node Models to Vaccines, Pathogens, and Drugs In Vitro

The abovementioned LNs chip devices successfully simulate multiple functions of natural LNs. However, they only summarize the selective characteristics of human LNs. Most lymph node chip studies have focused on simulating the behavior of immune cells, such as their response to chemokine gradients, migration, and interaction with other immune cells. As the primary function of LNs is to filter foreign particles, pathogens, and cancer cells, improved lymph node-on-chip devices should be able to simulate the immune response against these. Surprisingly, only a few studies have evaluated the ability of LNs to recognize pathogens and resist infection. Since the initial accumulation of immune cells and drugs usually occurs in LNs, an in vitro system that mimics the anatomy and physiology of LNs in vivo will allow the study of the mechanism of drug efficacy, efficiency and toxicity. This is essential for evaluating the toxicity of candidate drugs to immune components. Lymph node-on-chip can facilitate research into the interaction mechanism between immune cells, candidate drugs, and vaccine development.

Giese et al. developed HuALN (Human artificial lymph node), a membrane-based 3D bioreactor that mimics the structure of LNs [108]. This model allows long-term (14–30 days) repeated drug exposure to induce and monitor cellular and humoral immunity. The authors introduced peripheral blood mononuclear cells (PBMCs) and the commercial viral vaccine hepatitis A (Havrix^TM^) into the model. Quantitative analysis of the cytokine secretion spectrum showed that Havrix^TM^ induced an early pro-inflammatory response rapidly downregulated during the first days of culture. The addition of immunosuppressive drugs (dexamethasone) to the system significantly reduced the secretion of cytokines. Immune cells were then restimulated with free antigen and antigen-loaded DCs to induce the release of tumor necrosis factor-alpha (TNF-a), and T helper 2 cells promoted the release of the cytokines Interleukin-6 (IL-6) and Interleukin-10 (IL-10). In this model, plasma cells were regenerated, and the specificity of the plasma cells was demonstrated by antigen binding to the pp65 antigen. These results demonstrate HuALN’s ability to simulate immune responses against vaccines, pathogens, and drugs. Subsequent researchers used this model to test the immune effects of wild-type and fucosylated RSV-F-free glycoprotein vaccines. Additionally, HuALN can be a useful prediction tool for evaluating the immunogenicity of protein aggregates [109,110].

The critical structures of secondary lymphoid organs (such as LNs or the spleen) are primary LFs and GCs, especially during the “activated state” of inflammation or infection [108]. The formation of GCs indicates protective immunity against infections, which can predict the efficacy of vaccines and immunotherapies [111,112]. The enhanced lymph node-on-chip facilitates the study of the interaction mechanism between immune cells and drug candidates and vaccine development. It should reflect immune effects using cytokines and reveal lymph node-like biomarkers, the formation of neoplastic LFs or GCs, and the differentiation and development of plasma cells.

**Figure 6:**
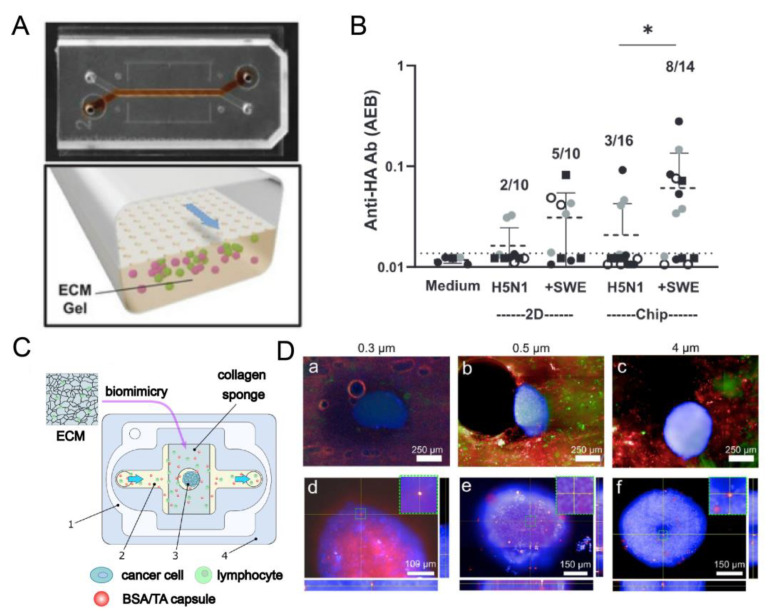
(**A**) Schematic diagram of the lymphoid follicle-like microfluidic chip device. (**B**) Anti-HA Ab signals were detected in unstimulated (medium) or H5N1 antigen (H5N1) alone or SWE (+SWE) stimulated LF Chip effluent or cell culture medium maintained in 2D culture (2D) for 9 days, which can be used to develop vaccines and adjuvant therapeutics. Each data point indicates one well or chip; different colored points indicate chips or wells from 4 independent donors. Statistical differences are tested using the Kruskal-Wallis test followed by pair-wise testing using the uncorrected Dunn’s test); * *p* < 0.05. [61]. Reproduced with permission from Girija Goyal, Advanced Science.; published by Wiley-VCH GmbH 2022. (**C**) Schematic of the chip used to study secondary tumors. (**D**) A total of 0.3, 0.5, and 4 μm bovine serum albumin (BSA)/tannic acid (TA) capsules were mixed with lymphocytes and pumped to the chip. Capsule penetration was examined using fluorescence chip scanning and quantitative image analysis. Lymphocyte cells (Calcein Am, green fluorescence), and (**a**) 0.3, (**b**) 0.5, and (**c**) 4 μm polymer capsules (Cy5, red fluorescence). (**d**–**f**) Orthogonal fluorescence images of cell spheroids with internalized (**d**) 0.3, (**e**) 0.5, and (**f**) 4 μm capsules [33]. Reproduced with permission from Sergei V. German, International Journal of Molecular Sciences; published by MDPI 2023.

Activation-induced cytidine deaminase (AID) and CXCL13 were expressed on the resulting 3D multicellular aggregates. The expression of AID demonstrated its ability to perform the follicular function that circulating B cells typically lack [113], and it mediates the crucial Ab switching reaction in the germinal center [114]. CXCL13 is also a critical condition for LF assembly [115] and is usually used as a biomarker for LF formation [111]. The expression of AID and CXCL13 confirms that LFs exist in the chip. Goyal et al. used primary human immune cells obtained noninvasively from peripheral blood to self-assemble a microfluidic organ chip of human lymphatic follicles (Figure 6A). Under perfusion conditions, the model did not exhibit the self-activation previously reported in high-density cultures of human B cells. When IL-4 and anti-CD40 Ab stimulated naïve B cells, IgG was detected in the chip effluent, confirming that B cells in the chip were functional and capable of class switching. From the 7th day, the formation of CD138^+^ plasma cells could be observed in the stimulated LF chip, simulating the development and differentiation of plasma cells in LF. In addition, the LF chip was inoculated with commercial fluzone influenza vaccines containing three different virus strains and H5N1 pandemic influenza antigen prepared with squalene oil in water adjuvant Squalenein-water emulsion (SWE). High levels of secreted anti-HA Ab (Figure 6B), increased production of antigen-specific IgG for plasma cell differentiation, and four cytokines (IFN-γ, IL-10, IL-2, GM-CSF) essential for T cell expansion, survival, and auxiliary functions were also detected. The text demonstrates that the chip can function as an LF chip, which is useful for investigating the underlying mechanisms of LF formation, physiology, and the human adaptive immune response. Additionally, it can be used to assess the effectiveness and safety of vaccines, adjuvants, and immunotherapeutic drugs in a patient-specific manner [61].

Furthermore, the Cesare team utilized a multi-chambered 3D LNs chip for drug response research. This device enables long-term cell culture and in situ viability testing, with the added benefit of measuring cell death without needing cell extraction. In their investigation of the immunomodulatory drug hydroxychloroquine (HCQ), the team found that HCQ reduced the migration rate of T cells and promoted continuous rotational movement (PRM). It was speculated that the alteration in rotational motion resulted from increased reactive oxygen species (ROS) production due to HCQ exposure. B cell motility and ROS levels were not significantly altered [59,60]. Cell movement is crucial in more complex immune responses, such as antigen presentation, cell activation, and Ab production [116,117]. The model allows the study of cell dynamics and complex biological processes and the evaluation of drug–cell interactions in multi-compartment and user-friendly devices. This may provide a novel, attractive platform for future vaccine and drug development. Ross and his team have designed a microfluidic chip that integrates mouse lymph node slices with a microfluidic system for localized chemical targeting. The three-layer PDMS microfluidic device consists of a perfusion chamber stacked on top of a stimulation port fed by an underlying microfluidic channel. Delivery of model therapeutic fluorescent dextran to specific lymph node regions demonstrated greater drug retention in the B cell zone than in the T cell zone. It provides a new platform for targeting and studying localized events in LNs and informs the development of new immunotherapies [118].

The LNs on biomimetic chips are useful for evaluating new immunotherapies’ immunotoxicity and solving existing drugs’ toxicity in clinical practice. Most organ-level lymph node models have focused on replicating humoral responses, specifically IgG secretion and germinal center development. In contrast, the model for organ-level development of the cytotoxic CD8^+^ T cell response is less developed, yet cytotoxic CD8^+^ T cells are critical for the induction of antiviral or antitumor immunity [119]. LN models are also needed to predict novel antigens’ immunogenicity following vaccination and assess humoral immunity, Ab production and B cell–T cell interactions [120,121]. Furthermore, vaccination and the adaptive immune response require integrating different lymphoid tissues. A more accurate lymphoid tissue model is needed to fully simulate the whole cascade of T/B cell generation, antigen presentation, and adaptive immune cell activation and migration [122].

### 3.2. Application of Lymph Node Model In Vitro in Disease and Cancer

The LNs organ chip is a powerful platform for studying immune-related diseases like cancer and inflammation [123,124]. Normal and diseased cells or tissues can be loaded onto a bionic LNs chip to monitor their behavior. A lymph node sinus microfluidic platform has been developed to simulate the hydrodynamic microenvironment of the lymph node subcapsular sinus. This device is designed to investigate the adhesion of cells related to lymph node metastasis in fluid flow after lymph node remodeling in response to disease or inflammation. Inflammation-induced remodeling caused changes in the fluid flow curve and the presence of adhesion ligands in the subcapsular sinus of LNs. Additionally, lymph-derived monocytes increase the adhesion of cancer cells in a lymph node flow-dependent manner, facilitating the invasion of cancer cells and monocyte immune cells during LNs metastasis (Figure 6B) [62].

Sergei et al. designed lymph node-on-chip (LNOC) as a tissue engineering model of secondary lymph node tumors formed due to metastasis (Figure 6C). The chip employs a collagen sponge to mimic the morphology and porosity of the ECM of human LNs, with a 3D sphere of 4T1 cells inside, simulating secondary tumors in lymphatic tissue. To prove the applicability of the chip in pharmacology, a mixture of bovine serum albumin (BSA)/tannic acid (TA) capsules and lymphocytes was pumped into the chip to evaluate the effect of the size of the contrast agent/drug carrier on the penetration and accumulation of particles in 3D spheres simulating secondary tumors. The fluorescence microscope scanning and quantitative image analysis results indicate that 0.3 μm-sized capsules can easily pass through the tumor sphere and penetrate the interior. This device can potentially become a reliable alternative to the early secondary tumor model and reduce the number of experiments in the preclinical research framework (Figure 6D). LNOC is also an attractive alternative for further analysis and understanding of the effectiveness of developed drug delivery carriers and contrast agents delivered to cells and organs [33].

Shim et al. reported a multi-compartment microfluidic chip co-culturing two tissue sections under continuous circulating flow to mimic tumor-induced immunosuppression. The device consisted of three layers of PDMS and a polycarbonate membrane containing two culture wells, two reservoirs, and two microchannels. Sections of mouse LNs were co-cultured with tumors or healthy tissues on the chip in the circulating medium, allowing soluble factors to be exchanged. Lymph node slices co-cultured with tumor slices were more susceptible to immunosuppression, suggesting that the chip could successfully mimic some features of tumor–immune interactions, such as T cell activation. In conclusion, this new microfluidic system provides an on-chip co-culture of paired tissue sections under continuous recirculation flow. It can mimic complex tumor–lymph node communication in vitro [88].

Lymph node-on-chip can aid researchers in comprehending the mechanism of the immune system in various diseases. By detecting and analyzing immune cell activity, protein interactions, gene expression, and other information, scientists can provide more accurate diagnoses and treatments for autoimmune and infectious diseases.

## 4. Conclusions and Perspectives

We summarize the design features of current microfluidic models to study LNs and discuss the use of these models in mimicking immune cell behaviors from immune cell motility to immune cell interactions and activation at the organ level, as well as the application of lymph node organs chips in immunology, drug screening, and vaccine development.

Central to the lymph node function is the unique spatial categorization of lymphocytes, stromal cells, and chemokines that drive the signaling cascade of the immune response. Specific messaging and collaboration between immune cells within the LNs allow for the timely triggering of reactive immunity in response to various pathologies [125]. Therefore, a significant challenge for lymph node chip models in simulating ECM is to reproduce the complex internal structure of LNs in vitro. The material of choice for lymph node chips is PDMS, usually fabricated using conventional soft lithography methods. Recently, 3D printing is emerging as an alternative to soft lithography, including inkjet, extrusion-based, light-based, and layer-by-layer high-precision build techniques for rapid prototyping of organs-on-a-chip. Cell-containing biomaterial models’ composition, spatial distribution, and structure can be precisely controlled [126,127]. This opens up the possibility of constructing lymph node chips with more complex structures. Electrospinning biofilm can replace synthetic PDMS or polycarbonate barriers used in microfluidic systems, with higher filtration performance and no need for additional coatings, providing a more natural microenvironment [128,129]. Electrospinning technology will also be helpful in the manufacture of complex models of LN biomaterials. More accurate modeling of in vivo tissue structures will not only deepen the understanding of lymph node immune cell movement and interactions across regions. Still, they will provide further insights into how drugs and vaccines flow, are delivered, and induce immune effects in the LNs.

Simulating the ECM in LNs on-chip not only presents challenges in terms of more complex physical cues but also requires the addition of biological cues to reproduce the ECM more realistically. LNs stromal cells are crucial for lymphocyte survival and migration, lymphatic transport, nutrient and antigen supply, immune monitoring, and adaptive immune response. They are critical for maintaining the structural integrity and function of LNs. Most lymph node chips currently use collagen or relocated protein-bound hydrogels as the matrix. In addition to the hydrogels mentioned above, they are covalently combining stromal cells [76] primary fibroblast reticulocytes (FRC) [77] or heparin [78] to mimic the ECM to promote the development and proliferation of immune cells. LNs stromal cells cannot be obtained from blood, so thymus and bone marrow stromal cells are often used instead of LNs stromal cells. There are multiple stromal cell subtypes in the LNs and most have not yet been obtained in engineered cultures, so the development of methods for reproducibly isolating, characterizing, and in vitro culturing specific LNs stromal cell subpopulations (e.g., FDCs) is vital to enable the construction of lymph node chips that more closely resemble the natural ECM environment in vivo [130]. These models can be used to study the complex interactions between stromal cells and other immune cells in immune cell chemotaxis and immune response.

As a platform for in vitro testing and screening of therapeutics, pharmacokinetics and toxicity are first tested in healthy tissue models [131]. Because genetic uniqueness may lead to different drug responses in different populations. Patient immune cells can be obtained through blood sampling or minimally invasive liquid biopsies and placed into a developed lymph node chips system to assess drug responses. For example, personalized chips are used to test the effectiveness of new vaccines developed against emerging influenza virus strains, drug resistance to cancer treatment, or to study the specific cellular response of specific populations to cancer treatment [132].

On the other hand, once the lymphatic vessels have transported the lymphatic fluid to the LNs, the fluid is filtered and returned to the bloodstream [133]. Lymphatic vessels play a vital role in immunity by facilitating communication between body tissues and lymphatic organs [134,135]. APCs and lymphocytes leave inflammatory tissues and enter nearby afferent lymphatic vessels, crucial for initiating and maintaining the immune response [136]. Most of the existing lymph node-on-chip channels are made and simulated using biomaterials. In the future, it is recommended that the lymphatic vessel and vascular model be integrated with the lymph node-on-chip. This will establish a lymphatic vessel lymph node vascular microcirculation organ chip, which can help to characterize the role of cell endothelial/matrix crosstalk and LNs microenvironment in immune response and barrier [137].

To date, some immune organ chips have been developed and their relevance to developing new drugs has been discussed [138,139]. However, the systemic effects of drugs cannot be studied with a single-organ chip. In an ideal immune chip system, candidate drugs would be introduced into the skin equivalent (similar to subcutaneous injection) or an intestinal equivalent (similar to oral administration). Subsequently, the drug will be transported to the spleen and LNs through the vascular and lymph vessels. The interactions of candidate drugs with the immune components in each organ will be accurately evaluated and quantified. This multi-organ chip technology can help scientists better understand disease pathogenesis and evaluate drug toxicity and effectiveness. It can also accelerate drug research and development, reduce the high cost and loss rate associated with drug development, and support the development of personalized and precision medicine. More biologically complex lymph node–multiorgan chip systems may become the next scientific frontier.

## Figures and Tables

**Table 1:** Summary of existing in vitro lymph nodes on-chip.

	Reference Article	Spatial Configuration	Lymph Node Scaffolding Material	ECM Material	Dynamic Condition	Fluid Flow Rate	Cellular Component	CELL Source	Cell Culture Time	Validated Immune System Function
1	Haessler et al. (2011) [52]	Immune cells in the middle channel and chemokine channels of different concentrations on either side.	Agarose	Hydrogel (Type I collagen+ Matrigel)	Active pumping	1 μL/min (chemokine)	Dendritic cell (primary)	Mouse	120 min	Establish stable and well-defined gradients in advance. Visualize live-cell migration. The DC migrated more efficiently to the higher gradient of CCL21.
2	Haessler et al. (2009) [53]	1.0 mm thick agarose membrane patterned with four sets of three-channel units, each unit containing a cell-ECM channel and two flow channels.	Agarose	Hydrogel (Type I collagen)	Active pumping	5 μL/min (chemokine)	Dendritic cell	Mouse bone marrow		Quantified the chemotactic response of murine DC to a gradient of CCL19
3	Ricart et al. (2011) [54]	Three different cytokine input mix regions (forms smooth gradient).	PDMS	Fibronectin	Syringe pump	9 μL/min (chemokine)	Dendritic cell	Mouse bone marrow	60 min	A single chemokine gradient and a competitive chemokine gradient were presented in the controlled microenvironment (CCL21, CCL19, CXCL12).
4	Moura et al. (2016) [55]	It has one main channel, two entrances, and two exits. Two distinct inlets pump CD4^+^ T cells and CD8^+^ T cells, respectively.	PDMS	Hydrogel (Collagen or Fibronectin)	Syringe pump	10^−4^–1 mL/min (T cell)	Murine tumor DC MF2.2D, OVAII RF33.70/OVAI	Mouse		Dynamic interaction of flowing lymphocytes with adherent DC, Effects of low and high shear stress variations on adhesion
5	Lin et al. (2006) [56]	A “Y” type fluidic channel.	PDMS	Fibronectin	Syringe pump	0.2 mL/min (chemokine)	T cell (activation)	Human Blood	20 min	Human T cells in response to single and competing gradients of chemokine CCL19 and CXCL12.
6	Mitra et al. (2013) [57]	Two layers of PDMS: the top layer contains the chemotaxis chamber, and the bottom layer includes the T cell compartment.	PDMS	Hydrogel	Syringe pump	0.4–0.5 μL/min	MUTZ-3: Human dendritic cell line T cell	Human Human Blood	2 h	Mature DCs are subject to a gradient effect by the chemokine CCL19 Mature DCs are collected in T cell compartments to induce T cell activation.
7	Sonmez et al. (2020) [58]	PC membrane filters separate two PDMS layers: the upper layer consists of flow channels, and the lower layer consists of flow-free chambers.	PDMS (0.4 μm PC membrane filter)	Fibronectin			Jurkat: Human T cell line	Human	30 min	The chemotaxis of the Jurkat cells was also found to be governed by the CXCL12 gradient and the average CXCL12 concentration.
8	Shanti et al. (2020) [59]	A multi-chamber bioreactor, separated by circularly distributed microcolumns. The outermost region corresponds to the subcapsular sinus, the middle region corresponds to the reticular ductal structure, and the inner region is divided into upper and lower regions, corresponding to the B follicle and paracortex.	PDMS (Hydrogel microcolumns)	Hydrogel (Type I collagen)	micropump		EB1: Human B cell line THP-1:Human Dendritic cell line Jurkat: Human T cell line	Human	72 h	Long-term culture and in situ viability testing of Sertoli cells Interactions between different cell types across chamber boundaries were observed. The flow pattern of lymphatic fluid was replicated.
9	Hallfors et al. (2021) [60]				microfluidic pump S	3 μL/min	Raji B: Human B cell line Jurkat: Human T cell line			Tested the effect of the immunomodulatory drug hydroxychloroquine (HCQ) on cells.
10	Goyal et al. (2022) [61]	Two channels are divided by a porous membrane; the lower channel consists of T, B lymphocytes, and hydrogels, and the upper channel is continuously perfused with medium	PDMS	Hydrogel	peristaltic pumps or Automated Zoe Organ Chip instruments	60 µL/h	T cell B cell	Human Blood	>9 d	Mimic germinal center formation, class switching, and Ab production. Antigen-specific Ab can be produced by the commercial Fluzone influenza vaccine for three different strains and the H5N1 pandemic influenza antigen inoculated LF chip formulated with the oil-in-water adjuvant SVE of squalene.
11	Birmingham et al. (2020) [62]	Constructed from a sheet of PDMS and a polystyrene tissue culture plate between which is a 125 μm adhesive gasket.	PDMS	No hydrogel	Active pumping	2.5 μg/mL	Thp1 human monocyte line LS174T human colon cancer cell line PANC-1 human pancreatic cell line	Human		Effect of subcapsular sinus biophysical (flow and structure) and biochemical (adhesion molecule expression) remodeling on cellular adhesion.
12	German et al. (2023) [33]	A central channel with an extension in the center of the main channel in which a collagen sponge is mounted, inside which cell spheroids are placed.	PDMS	Collagen sponge	Micro pumps	0.65 mL/h	4T1 breast cancer spheroids Jurkat cell	Human		To evaluate the effect of contrast/drug vehicle size on the penetration and accumulation of particles in 3D spheroids simulating secondary tumors with lymphadenopathy.

## Data Availability

Not applicable.
